# The added value of the visual analysis of DWI in post-surgery follow-up of soft tissue sarcoma of the extremities: do we really need ADC?

**DOI:** 10.1007/s11547-023-01613-w

**Published:** 2023-03-30

**Authors:** Virna Zampa, Giacomo Aringhieri, Rachele Tintori, Piercarlo Rossi, Lorenzo Andreani, Alessandro Franchi

**Affiliations:** 1 Diagnostic and Interventional Radiology, AOUP, Pisa, Italy; 2 Diagnostic and Interventional Radiology, Department of Translational Research and New Technology in Medicine and Surgery, University of Pisa, Pisa, Italy; 3 Orthopaedic and Traumatology Clinic, AOUP, Pisa, Italy; 4 Pathology Unit, Department of Translational Research and New Technology in Medicine and Surgery, University of Pisa, Pisa, Italy

**Keywords:** Soft tissue sarcoma, MRI, DWI, ADC, Recurrence, Post-surgery

## Abstract

**Introduction:**

MRI has a fundamental role in the follow-up of soft tissue sarcomas (STSs). However, the differentiation of recurrences/residual disease from post-surgical changes is a complex task, with a central role for the radiologist.

**Materials and methods:**

We retrospectively evaluated 64 post-surgery MRI for extremities STSs. MR protocol included DWI (*b* = 0, 1000). Two radiologists were asked to consensually evaluate: presence/absence of tumoral nodules, lesion conspicuity, imaging diagnostic confidence, ADC values, and DWI overall image quality. The gold standard was histology or MR follow-up.

**Results:**

Thirty-seven lesions in 29/64 patients were confirmed as local recurrence or residual disease (*n* = 16 ≤ 1 cm) with 1 MR false positive. On DWI, the conspicuity of the proved tumor lesions resulted excellent in 29/37, good in 3/37 and low in 5/37, higher than conventional imaging. A statistically significant higher diagnostic confidence of DWI compared to conventional imaging (*p* < 0.001) and DCE (*p* = 0.009) was observed. In the 37 histologically confirmed lesions, mean ADC value was 1.31 × 10^–9^ m^2^/s. Overall scar tissues mean ADC was 1.70 × 10^–9^ m^2^/s. DWI quality resulted adequate in 81% and unsatisfactory in 5%.

**Conclusions:**

In this highly heterogeneous group of tumors, the role of ADC seems to be limited. Based on our experience, looking at DWI images makes the lesions promptly and easily detectable. This technique gives less deceptive findings making the reader more confident in detecting/excluding tumoral tissue; the main drawback is the image quality and the lack of standardization.

## Introduction

In extremity soft tissue sarcoma (STS), the local recurrence correlates with increased incidence of distant metastasis and decreased survival [1, 2]; thus, the earlier the diagnosis and surgical resection, the better the outcome [3–5]. The depiction of recurrences and their differentiation from post-therapeutic changes is a complex task, with a central role for the clinical radiologist [4–6]. MRI represents the fundamental tool in the follow-up of STS after surgery [7]. There are different possible scenarios: post-surgical changes without neoplastic tissue, local recurrence of tumor, or residual tumor tissue due to inadequate resection in cases with inadequate surgical margins or without appropriate pre-operative imaging and diagnosis [8]. Both post-operative and radiotherapy changes interfere with the interpretation of imaging findings causing difficulty in recognizing the persistence or the recurrent tumor tissue [9, 10]. Edema, hematoma, granulation tissue, necrosis, abscess, lymphocele, recent, or fibrotic scar tissue in the surgical bed can mimic the tumor signal intensity or obscure the tumor persistence [9].

Diffusion weighted imaging (DWI) reflects the cellular content of a lesion; the high cellularity leads to a diffusion restriction and, consequently, to low apparent diffusion coefficient (ADC) values. Nowadays, this sequence is, or should be, always included in the oncologic musculoskeletal MR protocol because it provides diagnostic additional elements in a very short time. In the diagnostic phase, DWI increases the detectability of tumors with high cellularity and helps in the characterization of the biologic aggressiveness of the lesion in specific histologic types [11]. In the pre-surgery phase, it helps to assess the response to radiotherapy or chemotherapy by means of changes of ADC values [11]. In the post-surgery phase, it can help to detect residual tumor tissue differentiating it from post-operative changes [12, 13] since post-surgery alterations are supposed to have higher diffusivity than tumor tissue [11].

The aims of this retrospective study are to assess the diagnostic value of visual signal interpretation of DWI sequence and ADC calculation compared to conventional MRI and to determine the added value of DWI and ADC in the diagnostic performance of MRI in the follow-up of extremity STSs.

## Materials and methods

From June 2016 to January 2022, we retrospectively evaluated 64 consecutive patients with extremity STS who underwent post-surgical MRI for scheduled follow-up (*n* = 52) or because of Whoops surgery or with positive margins after planned excision (*n* = 12).

All examinations were performed with a 1.5 Tesla MR Scanner (GE Signa HDXT TwinSpeed—software v. 15.0) using the proper coil according to the anatomic region and clinical setting. The MR protocol included conventional imaging (T1 and T2 weighted, STIR, GRE) and DWI using *b* = 0, 1000, a relatively high *b* to minimize perfusion effect and mitigate the effects of “T2-shine through.”

Single-shot echo planar DWI was performed choosing the most appropriate plane and a field of view large enough to include the edges of the scar adjacent post-therapeutic alterations (15-42 cm) with the following parameters:

TR = 2800–7000 ms, TE = the lowest possible value (53-75 ms), matrix phase value = 256, matrix frequency value = 86–128, slice thickness = 3–5 mm, number of averages = 5–7 (for both *b* values: 0 and 1000), receiver bandwidth = 250 kHz, parallel imaging (ASSET; acceleration factor 2).

When the diagnosis of presence or absence of sarcomatous tissue was sure based on conventional and DWI, dynamic contrast enhancement (DCE) was not considered necessary. In 27/64 patients, a DCE study was performed (2D/3D fast SPGR with fat saturation; resolution time = 8–15 s).

### Image analysis

MRI examinations were evaluated in consensus by two radiologists with 26 and 7 years of experience in musculoskeletal imaging (*BLINDED* and *BLINDED*), respectively.

Image evaluation tasks are herein reported.

To detect the presence of residual tumor tissue/recurrence based on the lesion characteristics reported in Table 1.

**Table 1:** Image analysis details

*Lesion characteristics for suspected recurrent/residual STS*		
Morphology		Size (recording if smaller or bigger than 1 cm), nodular shape, margin
Conventional MR		Signal intensity (SI) characteristics on conventional imaging
DWI		Focal hyperintensity on DWI
DCE		Focal nodular lesion with early and rapid enhancement on DCE images
*Conspicuity (3-point scale)*		
1	Poor	Low visibility of the nodule, with slight SI abnormalities and poor visible contours
2	Good	Moderate visibility of the nodule with mild SI abnormalities and fair visible contours
3	Excellent	High visibility of the nodule with high SI abnormalities and clear visible contours
*Diagnostic confidence (3-point scale)*		
1	Low	Uncertain presence/absence of tumor
2	Mid	Possible presence/absence of tumor
3	High	Certain presence/absence of tumor
*Overall quality focusing on artifacts impact on DWI (4-point scale)*		
0	No	No artifacts, no impact on DWI quality
1	Low	Artifacts with low impact on DWI quality
2	Moderate	Artifacts with moderate impact on DWI quality
3	High	Artifacts with high impact on DWI quality

*STS* Soft Tissue Sarcoma; *MR* Magnetic Resonance; *DCE* Dynamic Contrast Enhancement; *SI* Signal Intensity; *DWI* Diffusion Weighted Imaging

To score the conspicuity per each identified lesion using a 3-point scale separately on conventional imaging alone (MRI conspicuity), on conventional imaging plus DWI (DWI conspicuity), and conventional imaging plus DCE when available (DCE conspicuity) (Table 1).

The diagnostic confidence in diagnosing or excluding residual/recurrent disease was separately scored per each examination using a 3-point scale for conventional imaging alone (MRI diagnostic confidence), on conventional imaging plus DWI (DWI diagnostic confidence), and conventional imaging plus DCE when available (DCE diagnostic confidence) (Table 1).

To rate the overall quality focusing on artifacts (recording if ferromagnetic or technical) and their impact on DWI sequences using a 4-point scale (Table 1).

To calculate ADC of a suspected lesion and of post-operative changes, a region of interest (ROI) was set by the radiologists on the highest signal intensity area on DWI as well as on the scar tissue using ADW^®^ post-processing software (General Electrics—software v. 4.3) according to lesion or scar dimensions.

To assess inter-reader reliability, a second blind evaluation of all the MRI examinations was performed by a third blind radiologist with 3 years of experience in musculoskeletal imaging (*BLINDED*). In this case, image analysis was limited to identification of suspected lesions (positive or negative MRI), assessment of their conspicuity and diagnostic confidence scores, and lesion ADC measurement.

### Gold standard references

Gold standard for patients with nodules suspected of local recurrence or residual disease of STSs was the histological evaluation after surgical excision. Patients with no suspected findings at MRI were confirmed as disease-free after at least one year of MR follow-up (performed every 3 months).

### Diagnostic accuracy

Based on the described gold standard references we calculated sensitivity, specificity, overall accuracy, positive (PPV), and negative predictive values (NPV), for conventional MRI.

We separately calculated sensitivity, specificity, overall accuracy, PPV, and NPV for DWI and ADC alone. For DWI, every nodular hyperintensity detected on visual inspection was considered as positive test. For ADC, every lesion with ADC ≤ to the mean ADC value (resulted from the histologically proven lesions) was considered as positive test.

## Statistics

For descriptive analysis, mean and standard deviation were provided for continuous variables, while proportions, mode, and median were reported for nominal/categorical variables. Normal distribution was tested for each continuous variable using Shapiro–Wilk test. The significance of differences between the diagnostic confidence of DWI, conventional sequences, and DCE was calculated using Related-Samples Wilcoxon Signed Rank Test. Considering only patients with histological diagnosis of recurrence/residual STSs, the significance of differences between the conspicuity of DWI, conventional sequences, and DCE and ADC lesion versus ADC scar was tested using Related-Samples Wilcoxon Signed Rank Test; on ADC values, the same test was repeated excluding sarcomas with myxoid and chondroid components, which represent a well-known limitation of DWI [14]. The significance of differences between ADC values of the histologically confirmed lesions and of all the scars (including positive and negative examinations) were calculated using independent Mann–Whitney U test. Statistical analysis was performed using SPSS v.26.0 software (IBM Inc., Armonk, New York). All reported *P* values were two-tailed, and *P* values of < 0.05 were deemed statistically significant.

Inter-reader reliability between expert radiologists’ consensus evaluation and the third less-experienced radiologist blind assessment was obtained by Cohen’s Kappa test for categorical variables, such as lesion conspicuity, MR examination diagnostic confidence, as well as for the final MRI diagnosis (positive or negative MRI for STS). Bland–Altman plot was used to evaluate the inter-reader reliability of quantitative data, meant as lesion ADC.

## Results

Study population included 64 patients, 35 males and 29 females, with age range 10–94 and mean age 52.36 years. Pre-operative histological diagnoses and anatomic sites of tumors are reported in Table 2.

**Table 2:** Histologic types, frequency, and sites of primary tumors

Histological type	Patient number (*n* = 64)	Site
Myxofibrosarcoma	12	Shoulder, arm, forearm (4), pelvis, thigh (3), knee, foot
Synovial sarcoma	10	Hand (3), pelvis, thigh, knee (3), leg, foot
Pleomorphic sarcoma	10	Shoulder (2), pelvis (4), thigh (4)
Extraskeletal myxoid chondrosarcoma	4	Thigh (2), knee, foot
Myxoid liposarcoma	5	Thigh (4), leg
Liposarcoma	4	Thigh (3), knee
Leiomyosarcoma	4	Thigh (3), leg
Pleomorphic liposarcoma	3	Thigh, Knee (2)
Myxoinflammatory fibroblastic sarcoma	2	Arm, foot
Spindle cell sarcoma	2	Shoulder, thigh
Kaposi sarcoma	1	Leg
Clear cell sarcoma	1	Foot
Solitary fibrous tumor	1	Thigh
Round cell myxoid liposarcoma	1	Thigh
Extraskeletal chondrosarcoma	1	Pelvis
Pleomorphic rhabdomyosarcoma	1	Thigh
Myoepithelial carcinoma	1	Thigh

Based on the diagnostic criteria mentioned in Materials and Methods section, a total of 38 lesions in 30/64 patients were diagnosed on MRI as local recurrence or residual disease of STSs (2 patients with 2 nodules and 3 patients with 3 nodules). One out of these 30 patients was diagnosed as positive on MRI (ADC 0.8 × 10^–9^ m^2^/s), but no recurrence or residual disease in the post-surgical specimen was observed, resulting in a false positive. The other 37/38 lesions in 29 patients were confirmed by post-surgery histopathological evaluation. Among these 29 patients, 9 cases were positive after Whoops or positive margin at surgery (solitary lesions), while in 20 cases a total of 28 lesions were diagnosed as local recurrence; the histologic diagnoses are reported in Table 3. Out of 37 histologically proven nodules, 16 lesions (43%) had the maximum diameter equal or smaller than 1 cm and 21 greater (57%). Particularly, the mean and the range of the recurrence’s maximum diameter were 24 mm and 3–100 mm, respectively.

**Table 3:** Histologic types, frequency, and sites of recurrence/residual tumors

Histological type	Patient number (*n* = 29)	Site
Myxofibrosarcoma	8	Forearm (4), thigh (3), knee
Synovial sarcoma	3	Hand (2), foot
Extraskeletal myxoid chondrosarcoma	2	Thigh (2)
Pleomorphic sarcoma	3	Shoulder, pelvis (2)
Myxoinflammatory fibroblastic sarcoma	2	Arm, foot
Spindle cell sarcoma	2	Shoulder, thigh
Kaposi sarcoma	1	Leg
Clear cell sarcoma	1	Foot
Pleomorphic liposarcoma	1	Knee
Rhabdomyosarcoma	1	Forearm
Myxoid liposarcoma	1	Thigh
Round cell myxoid liposarcoma	1	Thigh
Extraskeletal chondrosarcoma	1	Pelvis
Pleomorphic rhabdomyosarcoma	1	Thigh
Leiomyosarcoma	1	Thigh

Excluding Whoops cases, the mean, the mode, and the range of time between surgical tumoral excision and first diagnosis of STSs recurrence are 18.5, 5, and 2–66 months, respectively.

DWI conspicuity of the histologically proven lesions resulted excellent (score 3) in 29/37, good (score 2) in 3/37, and low (score 1) in 5/37. The conspicuity of the lesions on conventional MRI alone was scored as excellent in 18/37, good in 11/37, and low in 8/37. In the 14/37 confirmed nodules with post-contrast acquisition, the DCE conspicuity of the lesions was considered excellent in 7/14, good in 3/14, and low in 4/14.

DWI conspicuity resulted higher with a statistical trend, albeit not statistically significant, compared to conventional MRI (*p* = 0.069) and DCE (*p* = 0.099). Notably, the sample of conspicuity analysis was based only on confirmed lesions (37 lesions in 30 out of the total of 64 patients). For DCE, the evaluation included only the 14 nodules, in which it was performed.

Diagnostic confidence in detecting or excluding suspected lesions resulted:

- High (score 3) in 55/64 examinations, mid (score 2) in 4/64, and low (score 1) in 5/64 on DWI.
- High in 18/64, mid in 18/64, and low in 28/64 on conventional imaging.

Considering the cases in which DCE was performed, the diagnostic confidence in detecting or excluding suspected lesions resulted high in 13/27, mid in 5/27, and low in 9/27.

The mode of the diagnostic confidence score was 1, 3, and 3, while the median was 2, 2, and 3 for conventional, DCE, and DWI sequences, respectively. A statistically significant higher performance of DWI compared to conventional imaging (*p* < 0.001) and DCE (*p* = 0.009) was observed.

In the 37 histologically confirmed lesions, a mean ADC value of 1.31 × 10^–9^ m^2^/s (range 0.5–2.6; SD 0.579) was observed. Excluding lesions with chondroid and/or myxoid intralesional components (*n* = 19, 50%), mean ADC value resulted in 0.97 × 10^–9^ m^2^/s (range 0.5–1.5; SD 0.297). Considering all patients, mean ADC of the scar tissues was 1.70 × 10^–9^ m^2^/s (range 0.3–2.5; SD 0.377). In histologically confirmed STSs recurrences, mean ADC of the scar tissue was 1.73 × 10^–9^ m^2^/s (range 1.1–2.2; SD 0.341). Excluding the only false positive result, ADC values of the histologically proven lesions resulted significantly lower than the those of all the scars, considering both positive and negative patients (*p* < 0.001). ADC values of the confirmed STS nodules resulted significantly lower than ones of the corresponding scar tissue (*p* = 0.000254), and excluding chondroid and myxoid lesions (50%), the statistical significance between lesion and scar ADCs was even greater (*p* = 0.000196).

Considering all the examinations, artifacts had low impact on DWI (score 1) in 14, mild (score 2) in 9, and high (score 3) in 3 cases, respectively. No impact was observed in 38/64 examinations (60%). Mode and median score values for artifacts impact on DWI were both 0.

The sources of artifacts were ferromagnetic (50%) due to post-surgical changes or clips in 32/64 and technical (19%) due to complex anatomical districts in 12/64 examinations; no artifact was observed in 20/64 examinations.

Sensitivity of MRI in detecting/excluding local recurrences or residual disease of STSs was 100%, while specificity was 97%. Overall accuracy of MRI was 98.6% with a false positive rate of 3% and no false negative. PNV and PPV value resulted in 100% and 97%, respectively.

Considering the accuracy of DWI alone for STS nodules, the number of true positive (TP), false positive (FP), true negative (TN), and false negative (FN) were 38, 7, 28, and 0, respectively. Sensitivity of DWI alone in detecting/excluding local recurrences or residual disease of STSs was 100%, while specificity was 80%. Overall accuracy of DWI alone was 90.4% with a false positive rate of 20% and no false negative. PNV and PPV value was 100% and 84%, respectively.

Considering the accuracy of ADC alone for STS nodules, the number of TP, FP, TN, and FN was 21, 7, 28, and 17, respectively. Sensitivity of ADC alone in detecting/excluding local recurrences or residual disease of STSs was 55%, while specificity was 80%. Overall accuracy of ADC alone was 67% with a false positive and negative rate of 20% and 45%, respectively. PNV and PPV value was 62% and 75%, respectively.

Inter-reader reliability was good for conventional MRI conspicuity with a *κ* coefficient of 0.725 (*p* < 0.001) and even better for DWI conspicuity, with a *κ* coefficient of 0.755 (*p* < 0.001). Poor concordance was observed both for conventional MRI and DWI diagnostic confidence with *κ* = 0.432 (*p* < 0.001) and *κ* = 0.344 (*p* < 0.001), respectively. Inter-reader reliability was not statistically significant for DCE.

According to the image evaluation instructions described in materials and methods section, the level of agreement between the two blind imaging assessments was almost perfect for MRI diagnosis of recurrent or residual STS with a *κ* coefficient of 0.944 (*p* < 0.001).

Regarding ADC measurements, neither significant inter-rater variability nor systematic bias was observed.

## Discussion

MRI is the modality of choice in the follow-up of STSs [15]; indeed, it can depict a significant number of clinically undetectable local recurrences in STSs of the extremities [4, 5, 16]. Nevertheless, post-surgery and post-radiotherapy soft tissue changes are challenging for the radiologist because they make the image interpretation more difficult and time-consuming [9].

Unlike morphologic MR imaging sequences, DWI adds functional information about tissue composition without intravenous contrast media [17]. It is a functional technique that has been proven as a promising tool for several applications in musculoskeletal pathologies and, particularly, in oncology [11, 18]. To our knowledge, only a few studies evaluate the additional role of functional sequences such as DWI in the diagnosis of recurrent STSs [19].

The aim of this study was to investigate the diagnostic ability and the drawbacks of DWI, focusing on the visual analysis rather than on ADC values. Since the main goal was the visual inspection rather than the characterization, we chose only one and relatively high *b* value (*b* = 1000) that partially minimizes the T2-shine through effect while maintaining an acceptable SNR.

Potentiality of DWI in the surveillance for STSs recurrence has been investigated. In the literature, good results of MRI considering both contrast enhancement and DWI are reported [12, 20]. However, most of papers are mainly centered on ADC values results [20, 21], which are reported to increase the sensitivity and specificity of MRI in this clinical setting [20, 21].

**Fig. 1:**
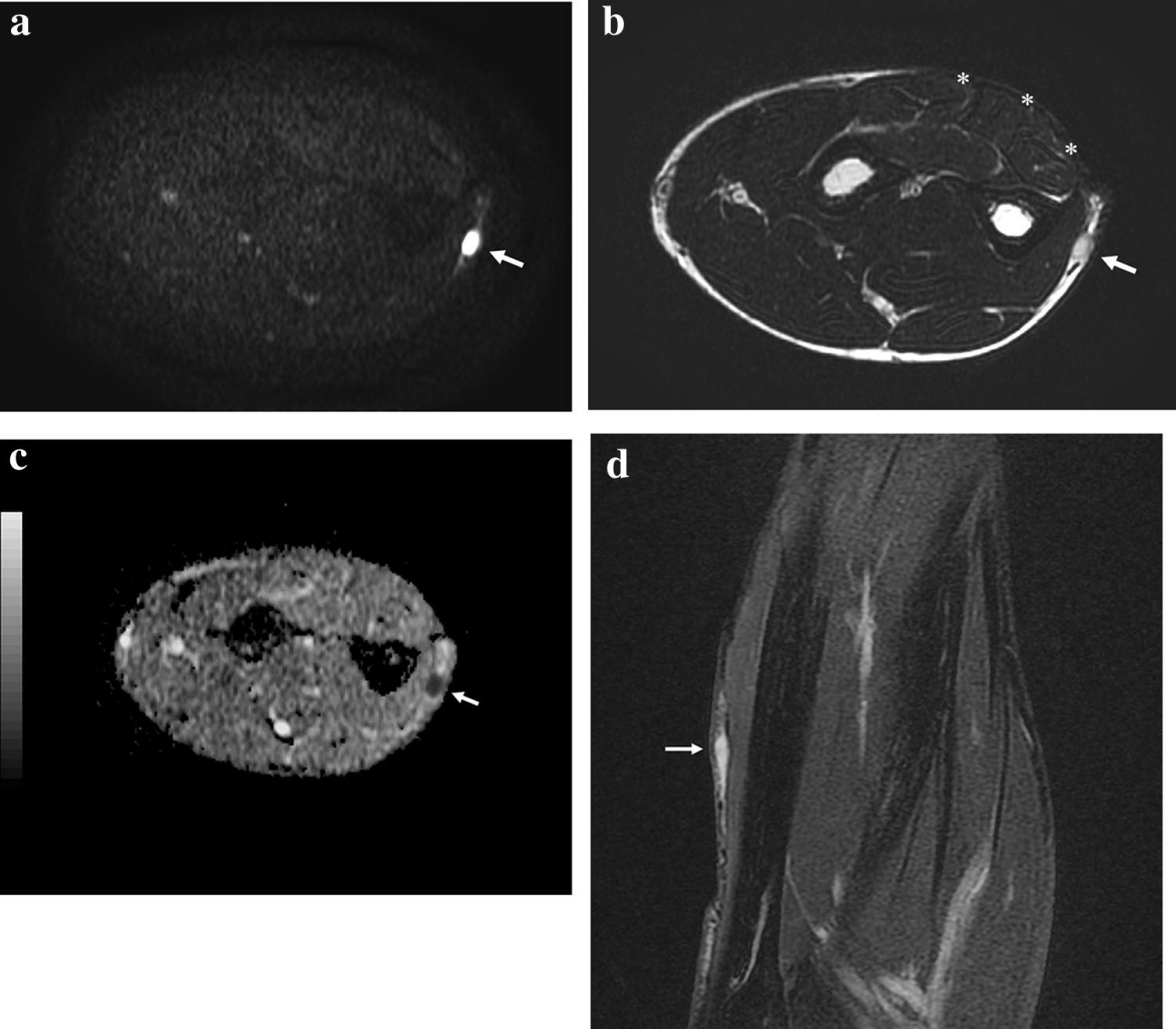
Example of the greater conspicuity of a lesion on DWI (*b* = 1000) compared to T2w and ADC map images. Fifty-one-year-old man with recurrent myxofibrosarcoma in the left forearm (**a**, **b**, **c**, **d**). Asterisks indicate scar tissue. **a** Axial DWI shows a small marked hyperintense nodule (*arrow*) in the subcutaneous fat with greater conspicuity compared to **b** T2w image, **c** ADC map, and fat-sat T2w image (**d**). Note the fibrous post-surgical scar tissue (*asterisks*) depicted on the T2w image

**Fig. 2:**
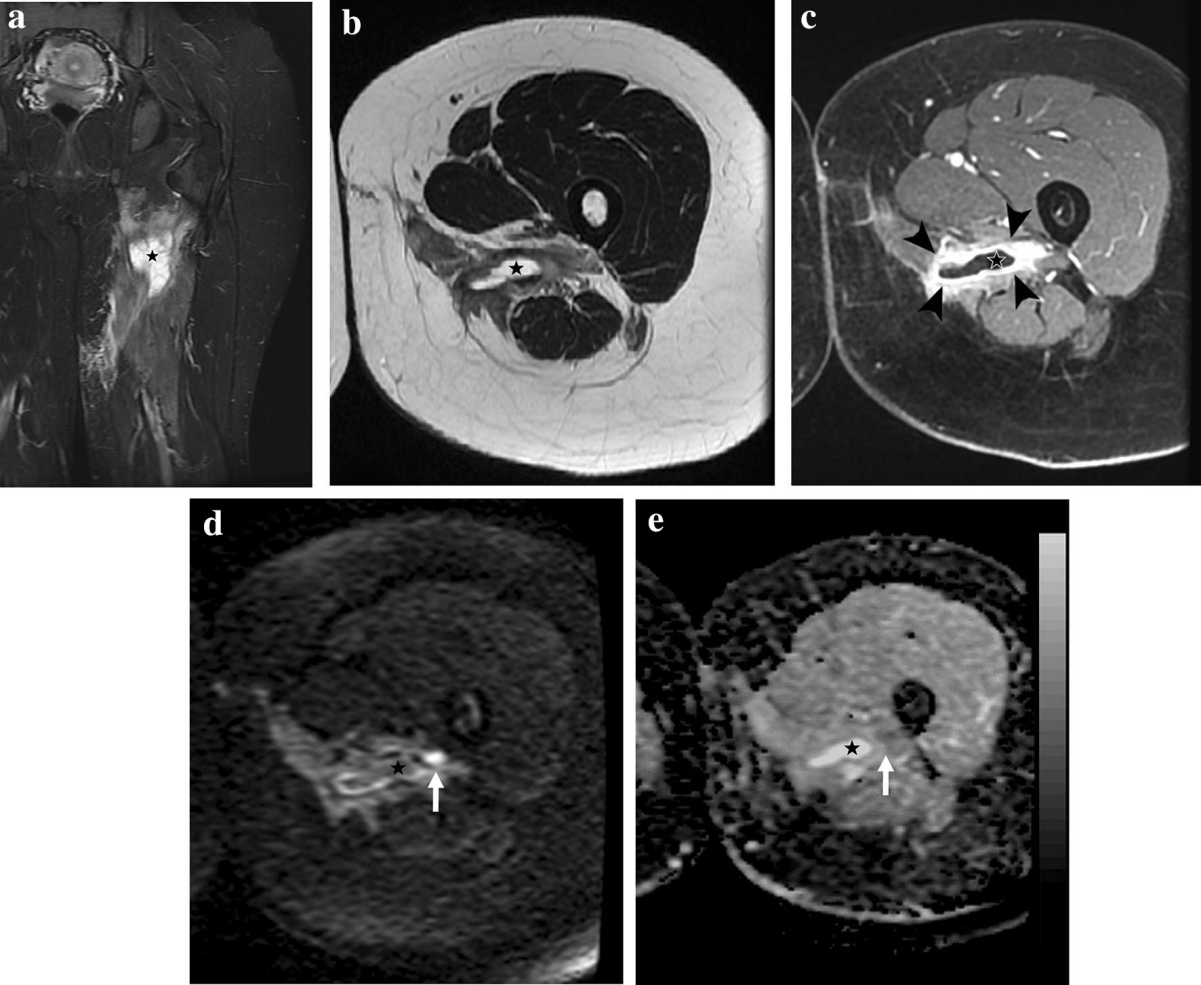
Example of the confounding aspect of the post-surgical changes on all the sequences except for *b* = 1000 DWI. Twenty-nine-year-old woman with residual spindle cell sarcoma in the proximal left thigh after surgical excision with positive margins. **a** Coronal STIR image showing diffuse soft tissue post-surgical edema with a large deep fluid collection (*star*). **b** Axial T2w and **c** axial post-contrast fat-sat T1w GRE images show thick and irregular enhancing tissue (*arrowheads*) at the periphery of the fluid collection (star). **d** Axial DWI at the same level reveals a small hyperintense nodule suggestive of residual disease. **e** The corresponding axial ADC map shows a barely visible focal hypointense lesion with an ADC value of 1.29 × 10^–9^ m^2^/s (ADC value of the scar 1.71 × 10^–9^ m.^2^/s)

In our study, diagnostic confidence of DWI was high with a median score of 3, significantly higher than conventional (median 2, *p* < 0.001) and DCE imaging (median 2, *p* = 0.009). These results are consistent with the expectations: On DWI, bright-restricted lesions obviously stand out against a dark background, showing higher detectability than on the other sequences (Fig. 1). Moreover, post-operative changes resulted less confounding on DWI with lesion visibility greater than on conventional MRI and DCE (Fig. 2). In fact, on DWI when a high *b* value is used, the post-surgery diffuse edema and subsequent diffuse edema-like pattern due to granulation tissue display a signal intensity markedly different from tumoral tissue (Fig. 2). In brief, the increased conspicuity of the lesions on DWI resulted in improved detection. This turned out to be particularly important in case of small lesions (16/37 lesions showed a maximum diameter ≤ 1 cm) because large nodules are obviously easily recognizable whatever the sequence. Diagnostic confidence resulted low in 44% for conventional imaging, 33% for DCE, and only in 8% for DWI. Particularly, conventional and DCE imaging resulted ambiguous in case of nodular shape of the scar mimicking an enhancing pseudomass [22].

**Fig. 3:**
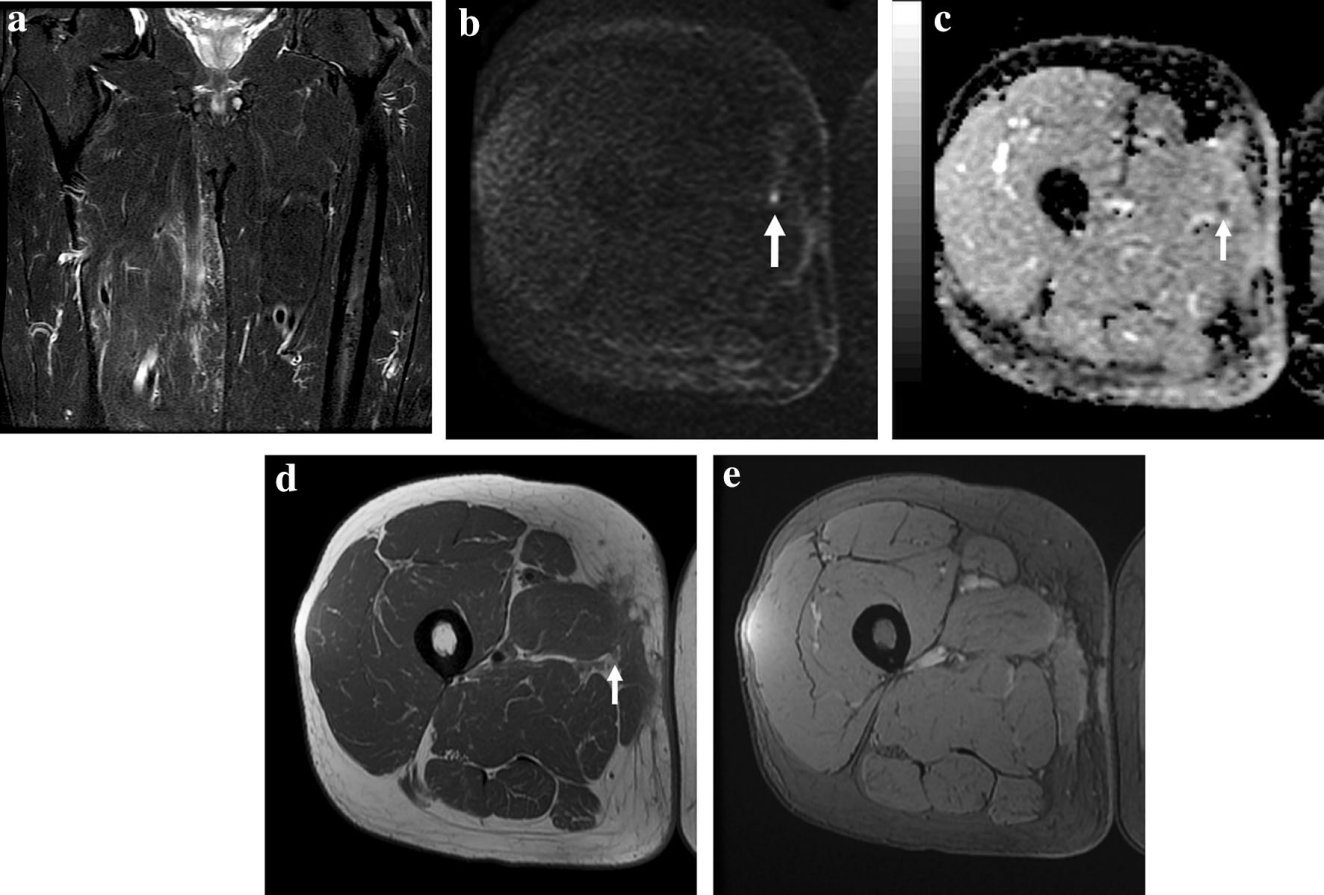
False positive case in which DWI findings were misinterpreted as small residual tumoral nodule. Fifty-three-year-old man MR follow-up after surgical excision of liposarcoma of the right thigh with positive margins. **a** Coronal STIR image shows diffuse soft tissue post-surgical edema of the medial proximal thigh compartment. **b** Axial DWI reveals a small hyperintense nodularity (*arrow*) within the scar, corresponding to a hypointense focal lesion on **c** ADC map (ADC = 0.8 × 10^–9^ m^2^/s). **d** Axial PDw image reveals a tiny nodule (*arrow*) within the intermuscular fat tissue corresponding to the DWI finding. **e** Axial T2*w GRE image at the same level shows no ferromagnetic artifacts

Among the 5 cases with low diagnostic confidence, DWI gave somewhat misleading findings in 3 cases. In one patient with early MRI due to positive margins after surgery, we observed a small bright nodule with low ADC value (0.8 × 10^–9^ m^2^/s) resulting in the only false positive case of our series (Fig. 3). This result did not change the outcome since the patient underwent radical resection. As known, in Whoops surgery, MRI is performed to determine the margins of the recent surgery rather than to detect tumor persistence, due to its insufficient accuracy, even if DCE is included [23, 24].

**Fig. 4:**
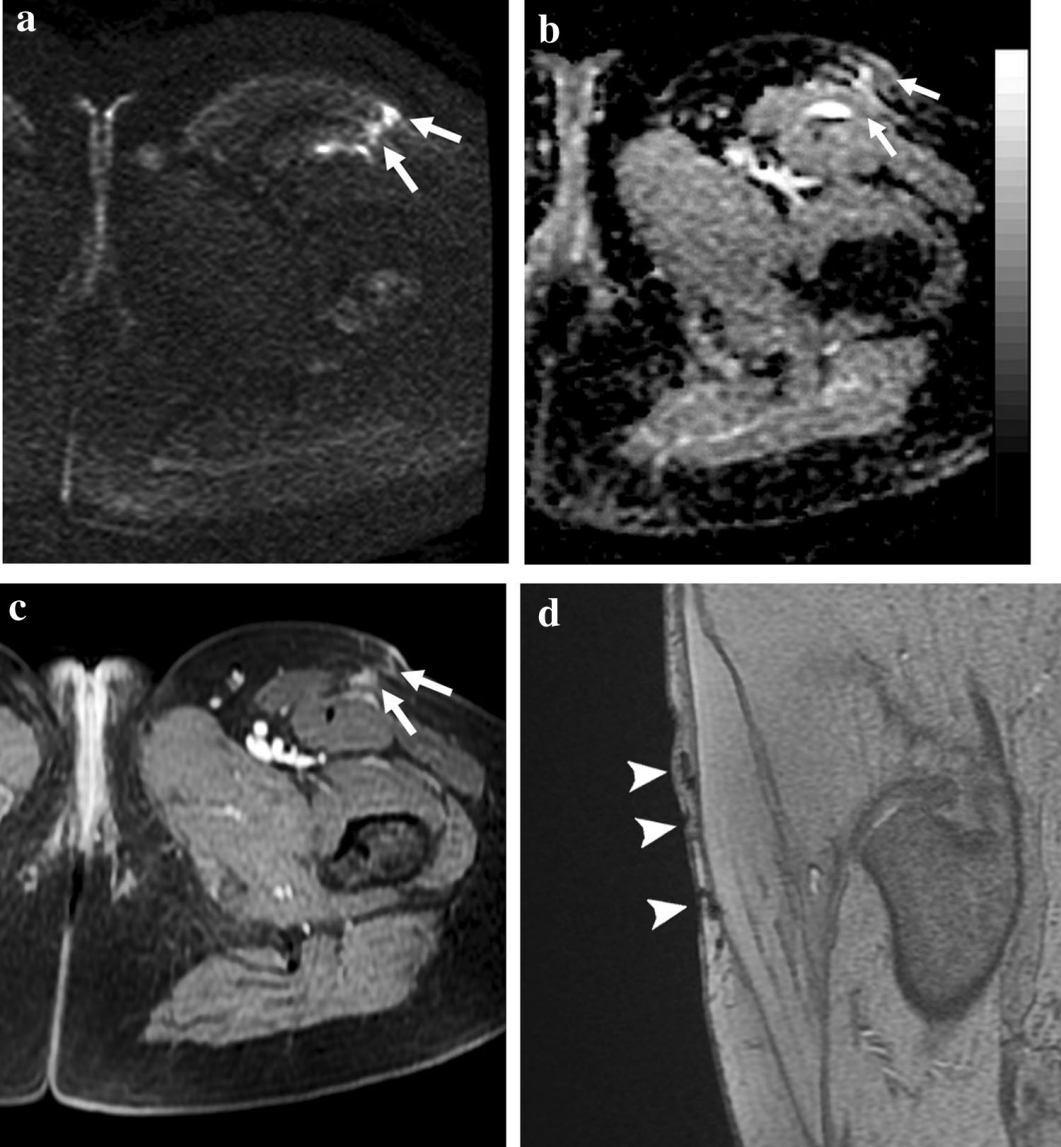
Effect of ferromagnetic artifacts on DWI. Forty-five-year-old woman MR follow-up after surgical excision of myxoid liposarcoma of the proximal left thigh. **a** Axial DWI showing tiny micronodular hyperintensities within the scar (*arrows*), corresponding to barely visible hypointensities on **b** ADC map. **c** Axial post-contrast fat-sat T1w GRE depicts a multinodular enhancement corresponding to DWI findings. **d** Sagittal T2*w GRE emphasizes the ferromagnetic nature of the artifacts (*arrowheads*)

**Fig. 5:**
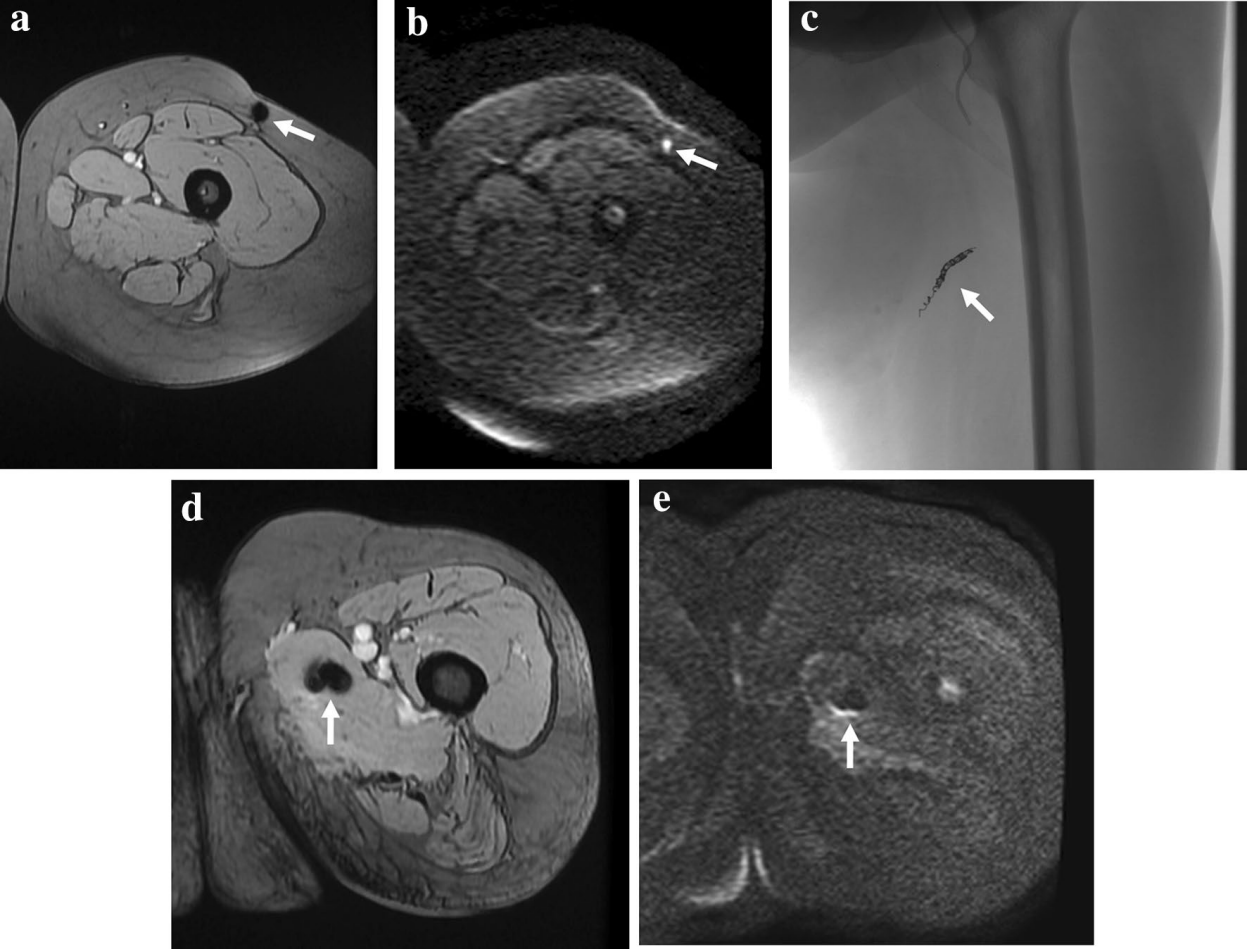
Two pictorial examples of ferromagnetic artifacts due to metallic objects of different sizes. Forty-year-old woman, MR follow-up after surgical excision of synovial sarcoma in the left thigh. (**a**, **b**); **a** Axial T2*w GRE shows a small artifact (*arrow*) due to metallic particles in the scar (blooming artifact). **b** Axial DWI shows a focal pseudonodular hyperintensity at the level of the metallic particle (*arrow*) without the typical signal void, possibly leading to diagnostic misinterpretation. Sixty-three-year-old woman, MR follow-up after surgical excision of pleomorphic sarcoma (**c**, **d**, **e**). **c** The fluoroscopic anteroposterior projection shows the presence of metallic spirals (*arrow*) due to pre-operative embolization. Axial **d** T2*w GRE and **e** DWI reveal the typical artifacts due to metallic spirals (*arrow*), emphasized by the T2* weighting

In two cases, we detected tiny multinodular contrast enhancement corresponding to hyperintense nodules on DWI; the ADC value was low (0.3 × 10^–9^ m^2^/s) in one case and high in the other one (1.63 × 10^–9^ m^2^/s). These findings caused a diagnostic dilemma, but since they were closely adjacent to ferromagnetic artifacts, identified on T2*w GRE, they were interpreted as scar tissue. As known, ferromagnetic artifacts appear as a signal void with peripheral hyperintensity (Fig. 4). When the source of artifacts has a certain dimension like in case of clips, they are easily recognizable. Contrarily, if only small metallic particles are left in the scar by surgical instruments, the interpretation can be more insidious on DWI because they can appear as focal pseudonodular hyperintensities (Fig. 5). This finding can be tricky, and therefore, we suggest to always include a T2*w GRE sequence (Figs. 4 and 5), where the typical artefactual signal void is emphasized and well recognizable. To our knowledge, this finding has never been described in the literature.

Regarding ADC quantitative analysis of our sample, the histologically proven lesions had significantly lower ADC values than the scars (mean ADC recurrences = 1.31 × 10^–9^ m^2^/s, mean ADC scar tissue = 1.73 × 10^–9^ m^2^/s). Our results differ from those of Del Grande et al. [21], who found average ADC of 1.08 and 0.9 × 10^–9^ m^2^/s for sarcomatous and scar tissue, respectively. To note, this study was performed on a 3 T scanner. The mean ADC values of recurrence and scar tissue found by Aktas et al. were 1.44 × 10^–9^ m^2^/s and 2.72 × 10^–9^ m^2^/s, respectively; the differences can be explained by the exclusion of fibrosis in the scar tissue ROI setting and only one case of myxoid sarcoma [25].

**Fig. 6:**
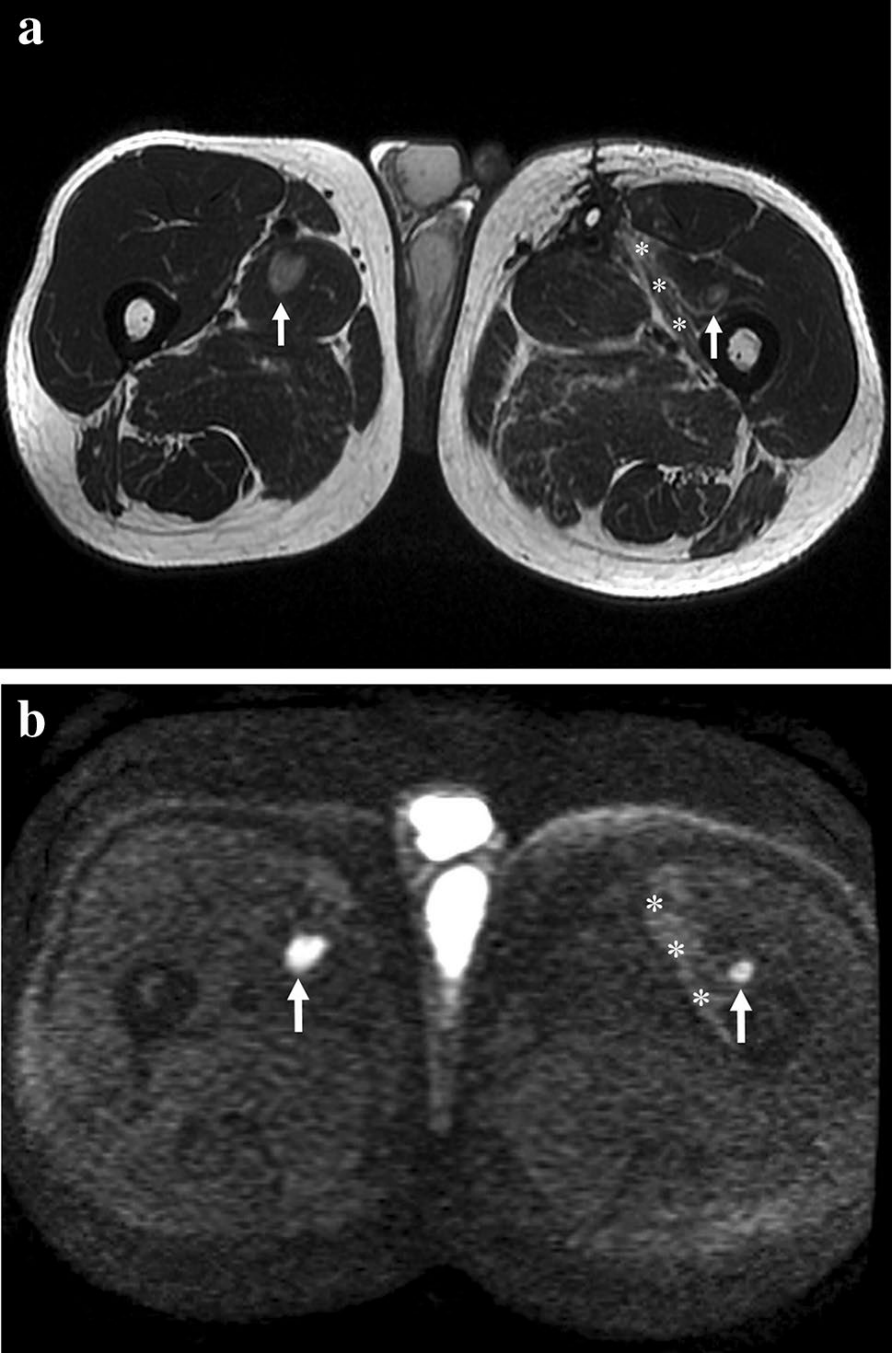
Example of the limits of the quantitative ADC analysis. Sixty-seven-year-old man with recurrent leiomyosarcoma of both proximal thighs. **a** Axial T2w image shows two hyperintense nodules (*arrows*) in the medial and anterior compartments of the right and left thigh, respectively. **b** Axial DWI confirms the two nodular lesions (*arrows*) exhibiting bright signal. ADC values of the right lesion (*arrow*) and the scar (*asterisks*) are 1.5 × 10^–9^ m^2^/s and 1.9 × 10^–9^ m^2^/s, respectively

In the specific scenario of STSs, limits of ADC are mostly related to chondroid and myxoid tumoral tissue [14] because they show high SI and high ADC values [18, 26, 27]. Actually, these components can be quite frequent, as in our study group (*n* = 33/64 patients—51.5%). In our series, excluding myxoid and chondroid sarcomas the mean ADC was 0.97 × 10^–9^ m^2^/s lower than the one reported by Eldaly et al. [20] (average ADC 1.3 × 10^–9^ m^2^/s). However, the ADC heavily depends on the tissue composition, and absolute values are susceptible to technique and equipment related variability. Other sarcomatous histotypes may show high ADC values; for example, in our series, one case of recurrent leiomyosarcoma showed an ADC of 1.5 × 10^–9^ m^2^/s (Fig. 6). This strengthens the concept of the poor reliability of ADC alone in this highly heterogeneous group of tumors.

**Fig. 7:**
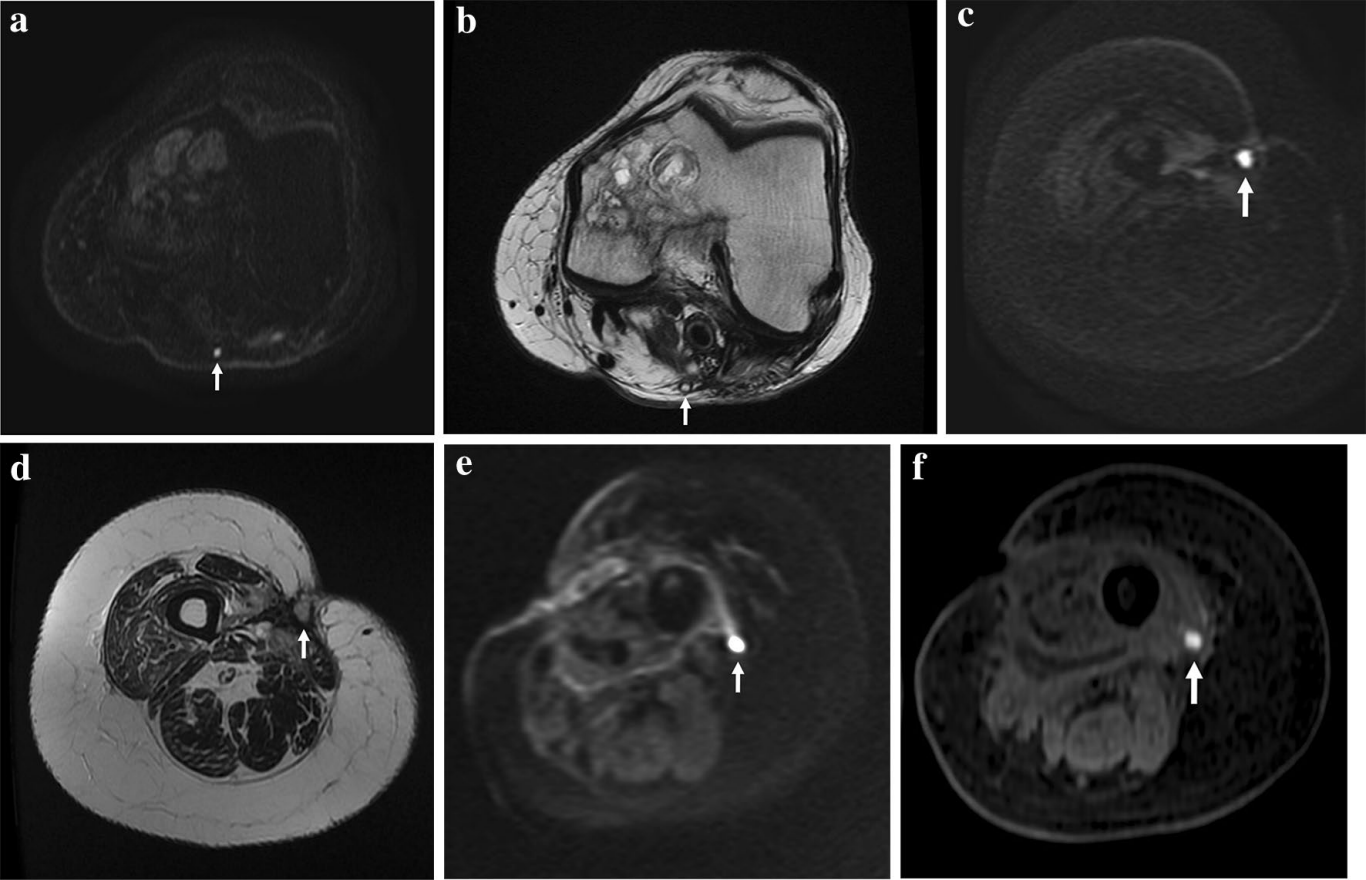
Examples of non-tumoral hyperintense nodules on DWI. Focally dilated vein (**a**, **b**); **a** axial DWI reveals a focal hyperintensity (*arrow*) posteriorly in the right knee with no diffusivity restriction (ADC = 1.31 × 10^–9^ m^2^/s). **b** axial T2w image shows a venous structure at the same level (*arrow*). Infective collection (**c**, **d**); **c** Axial DWI depicts a focal hyperintensity (*arrow*) at the level of the scar with diffusivity restriction (ADC = 0.7 × 10^–9^ m^2^/s). **d** Corresponding axial T2w image shows a subcutaneous infective collection (*arrow*) deeply to a cutaneous fistula. Intramuscular hematoma (**e**, **f**). **e** Axial DWI of the left thigh shows a focal hyperintensity (*arrow*) with diffusivity restriction (ADC = 0.44 × 10^–9^ m^2^/s). **f** Axial fat-sat GRE T1w shows a focal hyperintensity at the same level of DWI finding due to methaemoglobin (*arrow*)

High ADC can be related also to intratumoral necrosis, acellular regions, or low cellularity tumors [21]. Conversely, low ADC values can be encountered in case of non-neoplastic alterations, such as fibrosis, fat components, hemorrhage, and pus-like fluids (Fig. 7) [12].

Venous structures can appear bright on DWI (Fig. 7), but simply correlating them with the conventional sequences leads to the correct diagnosis. Bright lesions with low ADC values can be due to the high viscosity and debris of pus in infective collections [26] (Fig. 7) or caused by the methaemoglobin in hematomas and serous-hematic fluids (Fig. 7).

Surely, the ADC represents an additional diagnostic element although the simple DWI visual assessments resulted more useful in promptly detecting the presence/absence of tumoral tissue.

In fact, based on our data, DWI alone showed better results in terms of accuracy, sensitivity, PPV, and NPV values than ADC alone. For visual analysis we considered as positive test all the nodular hyperintensities (including venous structures, hematomas, serous-hematic, and purulent collections), thus limiting its specificity (Fig. 7). However, these findings are easily interpreted on conventional imaging.

**Fig. 8:**
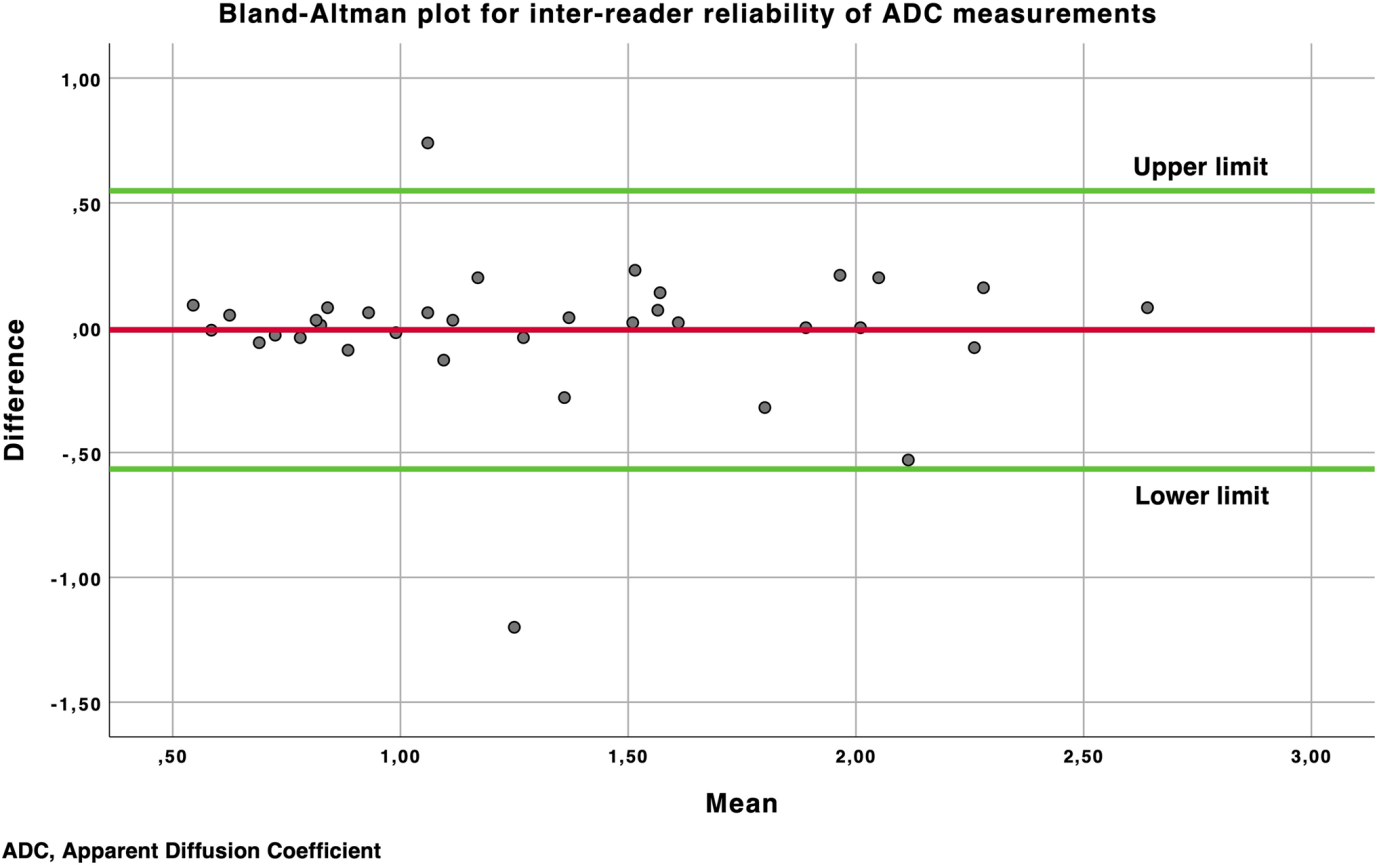
The Bland–Altman plot for the inter-reader reliability comparing high- and low-experienced radiologists shows a good reproducibility for ADC measurements

For ADC values alone, the accuracy remains limited mainly due to the high rate of chondroid and myxoid sarcomatous tissues. To summarize, although ADC values can be an additional diagnostic element, they cannot represent a reliable tool for confidently diagnose or rule out recurrences, despite its high reproducibility (Fig. 8).

The main drawback of DWI is of technical nature. This imaging technique shows an intrinsic poor signal-to-noise ratio and can be affected by several factors. Local magnetic field inhomogeneity and technical issues can remarkably affect image quality and, thus, the image interpretation. Artifacts are usually associated with motion (commonly seen in finger or toe) or positioning out of the isocenter of the magnet for practical issues (hand, elbow, shoulder), resulting in increased noise and inhomogeneous fat saturation. In our series, DWI quality was somehow disappointing particularly when small lesions were superficial and located in distal anatomic parts.

Ferromagnetic artifacts caused problems for the diagnostic interpretation only in the two cases (Fig. 4). However, DWI quality resulted adequate (score 0 and score 1) in 52 examinations (81%).

Some simple technical considerations can improve DWI quality. We always choose the highest bandwidth available (± 250 kHz) to minimize the chemical shift reducing the echo spacing and always prefer multi-channel coils implemented with parallel imaging. In fact, parallel imaging should be always adopted since it shortens echo-train lengths and reduces susceptibility and magnetic field inhomogeneity-related artifacts. According to the anatomic target, choosing small field of view and thin slice thickness is preferable to increase the spatial resolution, although a slice thickness lower than 3 mm usually results in poor image quality. Decreasing the size of the matrix frequency value yields a lower TE value: the lower the TE the lower the distortion of the image and the signal intensity loss with better SNR. Increasing the phase matrix value is useful for reducing the pixel size and thus the chemical shift. This is particularly important in the musculoskeletal imaging because of the frequent presence of large fat component. Finally, increasing the NEX would obviously provide a better SNR, but also increases the acquisition time.

This study has some limitations. First, the retrospective design can potentially affect patient homogeneity and lead to selection biases. Another limitation concerns the negative cases in which the reference standard did not include pathologic diagnosis for ethical reasons and relied only on follow-up. Then, the heterogeneity of the sample in the use of DCE can represent a limit. Finally, the sample size is relatively small due to the rarity of these tumors.

To sum up, our diagnostic strategy consists in carefully observing the b1000 DW images looking for something “bright” and then in correlating it with the anatomic images. We prefer to look at DWI rather than the ADC map because bright lesions stand out against the dark background, making them more promptly and easily detectable. When a “bright” lesion is evident on DWI and considered suspect for sarcomatous tissue, the analysis from qualitative shifts to quantitative to verify the real diffusion restriction of the lesion. We calculate the ADC value keeping in mind the above-mentioned pitfalls. We strongly suggest including a T2*w GRE sequence in the MR protocol to better identify ferromagnetic artifacts, avoiding possible DWI misinterpretation. When MRI findings are unequivocal and the quality of DWI is good enough, we skip the DCE study, reducing patient’s discomfort and risks, and saving time and money. Based on our results, the high sensitivity (100%) and negative predictive value (100%) of visual inspection of DWI allow us adopting this behavior also in negative cases, when no focal hyperintensity on DWI is observed.

Although the specific experience in the field of STSs can affect the diagnostic confidence of low-experienced radiologists, the inter-reader reliability resulted good for DWI conspicuity and almost perfect for MRI diagnosis. This demonstrates that the results obtained with this approach are highly repeatable, supporting the pivotal diagnostic role of DWI visual analysis.

In conclusion, DWI represents a promising diagnostic tool able to improve MRI detection of recurrent or residual tumor after surgery of STSs. Obviously, DWI findings always need to be correlated with conventional imaging to be properly interpreted.

Since image quality is the main drawback of this sequence, technologic advancements aimed at improving its performance are desirable to further increase DWI robustness as well as protocol standardization. Vendors should focus their research on further development to improve the quality of this sequence, measurement, and analysis methods for better repeatability and reproducibility. Further studies are needed to confirm and improve these results.
